# Früh beginnende Demenzen

**DOI:** 10.1007/s00115-020-00967-0

**Published:** 2020-07-28

**Authors:** Matthias Pawlowski, Andreas Johnen, Thomas Duning

**Affiliations:** Klinik für Neurologie mit Institut für Translationale Neurologie, Universitätsklinikum Münster, Albert-Schweitzer-Campus 1, Gebäude A1, 48149 Münster, Deutschland

**Keywords:** Präsenile Demenzen, Sekundäre Demenzen, Frontotemporale Lobärdegeneration, Alzheimer-Krankheit, Vaskuläre Demenz, Early-onset dementia, Secondary dementia, Frontotemporal lobar degeneration, Alzheimer disease, Vascular dementia

## Abstract

**Hintergrund:**

Aufgrund des demographischen Wandels sind Demenzen ein häufiger und dramatisch zunehmender Grund für ärztliche Vorstellungen. In etwa 8 % der Fälle treten sie bereits vor dem 65. Lebensjahr auf. Gerade bei jüngeren Patienten sind die psychosozialen und ökonomischen Folgen oft gravierend. Die Behandler stehen vor großen diagnostischen Herausforderungen. Eine rasche Diagnose ist für das Patientenmanagement von zentraler Bedeutung.

**Ziel der Arbeit/Fragestellung:**

Dieser Übersichtsartikel stellt die Besonderheiten der Demenzen bei jüngeren Menschen sowie die wichtigsten zugrunde liegenden Krankheitsbilder vor und vermittelt ein strukturiertes klinisch-diagnostisches Vorgehen.

**Methoden:**

Narrativer Review. Die Literatursuche wurde in PubMed durchgeführt.

**Ergebnisse:**

Das differenzialdiagnostische Spektrum von Demenzen bei jüngeren Menschen vor dem 65. Lebensjahr ist sehr breit. Die häufigsten Ursachen stellen die Alzheimer-Krankheit mit typischen oder atypischen klinischen Präsentationen sowie die frontotemporale Lobärdegeneration dar. Je jünger das Erkrankungsalter, desto höher ist der Anteil an behandelbaren und potenziell reversiblen Ursachen eines demenziellen Syndroms.

**Diskussion:**

Die Diagnostik primär neurodegenerativer Erkrankungen hat sich zunehmend verbessert, insbesondere unter Berücksichtigung einer stetig steigenden Zahl an klinischen, molekularen und bildgebenden Biomarkern. Dennoch muss die Diagnostik der Demenzen mit frühem Erkrankungsbeginn hypothesengeleitet erfolgen, d. h. nach einer präzisen klinisch-syndromalen Zuordnung der Symptome. So können unnötige und belastende Untersuchungen vermieden werden.

## Abstract (en)

**Background:**

Due to the demographic change dementia is a common and dramatically increasing reason for medical presentations. In approximately 8% of cases dementia occurs before the age of 65 years. The psychosocial and economic consequences are often severe, particularly in younger patients. Clinicians face major diagnostic challenges. A rapid diagnosis is crucial for patient counselling and management.

**Objective:**

This review article presents the special features of dementia in younger people, the most important underlying diseases and a rational clinical diagnostic approach.

**Methods:**

Narrative review. The literature search was carried out in PubMed.

**Results:**

The differential diagnostic spectrum of dementia in younger people under the age of 65 years is very broad. The most common causes are Alzheimer’s disease with typical or atypical clinical presentations and frontotemporal lobar degeneration. The younger the age of onset, the higher the proportion of treatable and potentially reversible causes of dementia.

**Conclusion:**

The diagnostics of primary neurodegenerative diseases have continuously improved, especially due to the availability of an increasing number of clinical, molecular and imaging biomarkers. Nevertheless, in order to avoid unnecessary and burdensome examinations, the diagnostic work-up of young onset dementia must be hypothesis-driven, i.e. following a precise clinical syndromic classification of the symptoms.

## Hintergrund

Demenzen sind für betroffene Patienten und ihre Angehörigen in der Regel mit erheblichen medizinischen, prognostischen und sozioökonomischen Folgen verbunden. Unsere Gesellschaft stellen sie vor große gesundheitsökonomische und gesellschaftspolitische Probleme [1]. Trotz der zunehmend gesünderen Lebensführung und der damit assoziierten Risikoreduktion, an einer Demenz zu erkranken, ist es aufgrund des demographischen Wandels in den vergangenen Jahrzehnten zu einem rasanten Anstieg der Anzahl an Patienten mit einer Demenz gekommen. Diese Entwicklung wird sich in den kommenden Jahrzehnten fortsetzen [1, 8]. Demenzen stellen typischerweise Erkrankungen des höheren Lebensalters dar; das Alter ist der bedeutendste Risikofaktor für die Entstehung einer Demenz. Die Alzheimer-Krankheit ist sowohl bei älteren als auch bei jüngeren Patienten die mit Abstand häufigste Ursache einer Demenz. Die jährliche Inzidenzrate einer Demenz steigt von etwa 0,5 % im Alter von 65 bis 70 Jahre auf 6–8 % bei Menschen >85 Jahre [1, 29]. Dennoch ist es wichtig, Demenzen und neurodegenerative Erkrankungen auch bei jüngeren Patienten als Differenzialdiagnose bei alltagsrelevanten kognitiven Defiziten zu bedenken, da sich etwa 8 % aller Demenzen vor dem 65. Lebensjahr manifestieren [10]. Diagnostik und Management von jüngeren Patienten mit demenziellen Syndromen stellen im klinischen Alltag große Herausforderungen dar, da deren Epidemiologie und klinische Präsentation in unterschiedlichen Altersgruppen erheblich variieren. Zudem ist das differenzialdiagnostische Spektrum bei Jüngeren deutlich breiter [10, 31]. In diesem Artikel geben wir einen Überblick über die häufigsten Ursachen von Demenzen bei Patienten <65 Jahre und vermitteln ein alltagspraktisches und standardisiertes diagnostisches Vorgehen.

## Definitionen und Epidemiologie

In der aktuellen S3-Leitlinie wird der Begriff Demenz – angelehnt an die ICD10-Klassifikation – klinisch definiert, d. h. als eine progrediente kognitive Störung mit alltagsrelevanter Ausprägung. Nach dem Feststellen dieses Symptomkomplexes wird eine weiterführende Diagnostik empfohlen, um die Ätiologie der Symptome festzustellen. Nicht neurodegenerative Erkrankungen sollten als Ursache ausgeschlossen werden. In der Revision der Leitlinie aus dem Jahr 2016 wird jedoch schon auf die aktuellen Entwicklungen der internationalen Diagnosekriterien hingewiesen, insbesondere auf die Sensitivität spezifischer laborchemischer Biomarker, wie z. B. Amyloid- oder Tau-Protein-Werte. Zudem werden die Erkrankungen als fortschreitendes Kontinuum betrachtet, deren Pathophysiologie sich langsam-progredient und meist schon Jahre oder Dekaden vor Symptombeginn zeigt, d. h. eine Unterteilung in z. B. Alzheimer-Demenz oder klinische Vorstufen findet nicht mehr statt, sondern nur noch die Definition Alzheimer-Krankheit.

Anhand des Manifestationsalters werden Demenzen recht willkürlich bzw. vorwiegend aus psychosozialen Gründen dichotomisiert in solche, die vor oder nach dem 65. Lebensjahr auftreten, wobei sich in der englischsprachigen Literatur hierfür die Begriffe „young onset dementia“ bzw. „late onset dementia“ etabliert haben [19]. Die früher verwandten Begriffe „präsenil“ bzw. „senil“ sollten heute keine Anwendung mehr finden, da sie suggerieren, dass alltagsrelevante kognitive Störungen bei älteren Menschen eine normale Entwicklung sind. Früh beginnende Demenzen umfassen daher per definitionem alle Demenzen mit einem Erkrankungsalter zwischen dem 18. und 65. Lebensjahr.

**Figure 1:**
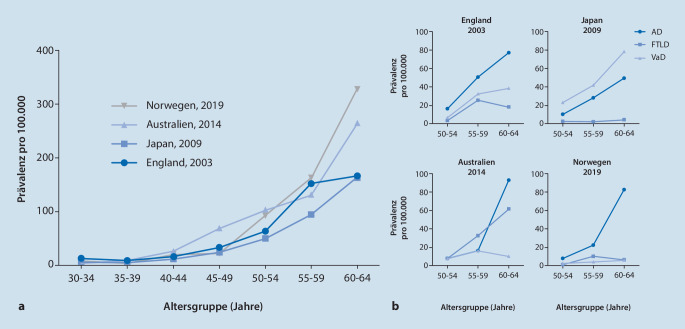

Es existieren kaum breit angelegte, epidemiologische Studien zur Prävalenz und Inzidenz früh beginnender Demenzen. Die untersuchten Fallzahlen sind klein und die eingeschlossenen Altersgruppen, Populationen, Methoden und Diagnosekriterien in den jeweiligen Studien deutlich verschieden [21]. In den vier größten, bevölkerungsbezogenen Registerstudien betrug die Prävalenz bei den 45- bis 64-Jährigen 81–143/100.000 Einwohner [14, 16, 20, 35], wobei sich eine altersabhängige exponentielle Zunahme der Prävalenz zeigte (Abb. 1a). Das durchschnittliche Manifestationsalter aller Demenzen, die sich vor dem 65. Lebensjahr manifestieren, beträgt 58 Jahre [10, 31]. Obwohl Demenzen bei älteren Menschen deutlich häufiger sind, macht der Anteil jüngerer Patienten in Gedächtnisambulanzen und spezialisierten Kliniken bis zu einem Drittel der Fälle aus [4].

## Das ätiologische Spektrum früh beginnender Demenzen

Die Ursachen der Demenzen können grob unterteilt werden in primär neurodegenerative Erkrankungen, vaskuläre Demenzen und das breite Spektrum der sekundären Demenzen. Die primär neurodegenerativen Erkrankungen sind nicht nur bei älteren Patienten, sondern auch bei Betroffenen mit Manifestationsalter zwischen dem 35. und 65. Lebensjahr die häufigste Ursache einer Demenz [18, 20, 21, 35]. Innerhalb dieser Gruppe stellt die Alzheimer-Krankheit die häufigste Ursache dar, gefolgt von der frontotemporalen Lobärdegeneration (FTLD) (Abb. 1b). Die Lewy-Körperchen-Demenz und Parkinson-Demenz, die bei älteren Patienten die zweithäufigste Ursache einer Demenz darstellen, sind bei Patienten vor dem 65. Lebensjahr hingegen seltener. Die vaskuläre Demenz wird formal nicht zu den sekundären Demenzen gezählt. Ihre Häufigkeit bzw. ihr Anteil an den Ursachen früh beginnender Demenzen unterliegt möglicherweise starken geographischen Schwankungen und war in einer japanischen Studie deutlich größer als in den europäischen/australischen Studien (Abb. 1b; [14, 16, 20, 35]).

Bei Patienten mit sehr früher klinischer Manifestation, d. h. vor dem 35. Lebensjahr, fallen die primär neurodegenerativen Erkrankungen proportional kaum ins Gewicht, stattdessen finden sich prozentual deutlich häufiger sekundäre Demenzen, also neurokognitive Störungen z. B. infolge metabolischer, genetischer oder inflammatorischer Erkrankungen [18]. Ab dem 35. Lebensjahr gleichen sich die unterschiedlichen Ursachen und deren relative Häufigkeit zwischen Demenzen bei jüngeren (<65 Jahre) und älteren (≥65 Jahre) Menschen zunehmend an [18, 31]. Umgekehrt gilt: Je früher das Manifestationsalter einer Demenz, desto eher handelt es sich um eine sekundäre Demenz [18, 31]. Sekundäre Demenzen sind bei jungen Menschen nicht nur prozentual deutlich häufiger als bei älteren Menschen, sondern das Spektrum der Differenzialdiagnosen ist auch wesentlich breiter (Tab. 1; [10, 31]).

### Alzheimer-Krankheit

Die Alzheimer-Krankheit manifestiert sich in unterschiedlichen klinischen Syndromen, welche alle durch die gleiche zugrunde liegende Neuropathologie gekennzeichnet sind: extrazelluläre Plaques aus Ablagerungen des Peptids Amyloid‑β (Aβ), intrazelluläre Ablagerungen des mikrotubuliassoziierten Proteins Tau und Neuroinflammation [34].

**Figure 2:**
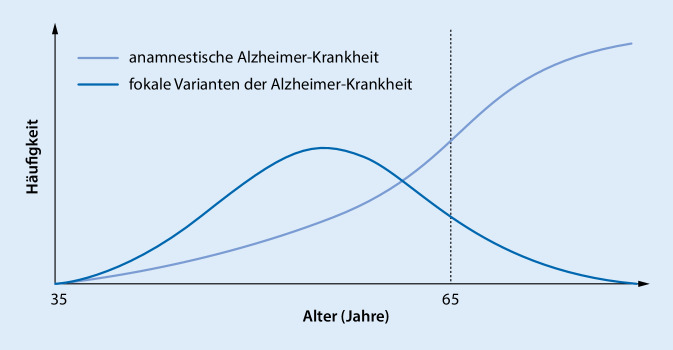

Bei der typischen Form der Alzheimer-Demenz, die sich in etwa 95 % der Fälle jenseits des 65. Lebensjahres manifestiert [1], stehen initial mnestische Störungen im Vordergrund, genauer eine Störung der Lern- und Merkfähigkeit, ohne oder nur mit geringem Profit durch Abrufhilfen. Im Gegensatz dazu, manifestiert sich die Krankheit bei jüngeren Patienten in etwa 20–65 % der Fälle in Form atypischer, fokaler Varianten (Abb. 2; [23]): Die zwei häufigsten Formen sind die posteriore kortikale Atrophie (PCA) und die logopenische Variante der primär progressiven Aphasie (PPA). Seltener ist die frontale Variante der Alzheimer-Krankheit [34].

Bei der PCA kommt es zu vorwiegend visuellen Symptomen trotz intakter primärer visueller Verarbeitung, d. h. das Sehen ist unbeeinträchtigt, jedoch ist die Interpretation der Seheindrücke gestört [7]. Im Vordergrund steht somit eine komplexe visuell-perzeptive kognitive Störung mit Simultanagnosie, okulärer Apraxie, optischer Ataxie und visueller Agnosie [7].

Bei der logopenischen Variante der primär progressiven Aphasie (lpPPA) handelt es sich um eine zunächst isoliert auftretende Sprachstörung, charakterisiert durch Wortfindungsstörungen, eine reduzierte Sprachproduktion bei relativ gut erhaltener Phonologie und Syntax sowie ausgeprägten Störungen des *verbalen* Kurzzeitgedächtnisses, welches sich insbesondere durch fehlerhaftes Nachsprechen mehrsilbiger Wörter manifestiert [13, 24].

Die frontale Variante der Alzheimer-Krankheit (auch als dysexekutive oder behaviorale Variante bezeichnet) weist klinisch zahlreiche Überschneidungen mit der behavioralen frontotemporalen Demenz auf: Sie ist durch eine frühzeitige Apathie und verminderte Initiierung produktiven Verhaltens (Apathie) gekennzeichnet, aber auch durch Disinhibition und Impulsivität [26].

### Frontotemporale Lobärdegeneration

Die frontotemporale Lobärdegeneration (FTLD) ist ein spezifischer neuropathologischer Begriff für primär neurodegenerative Erkrankungen mit Atrophie frontaler und/oder temporaler Strukturen sowie histologisch nachweisbaren, intrazellulären Ablagerungen aberranter Formen der Proteine Tau, TDP-43 oder FUS [2]. Das klinische Korrelat der FTLD ist in den meisten Fällen eine frontotemporale Demenz (FTD): Hierzu zählen die Verhaltensvariante der FTD (bvFTD) und die Sprachvarianten, also die verschiedenen Subtypen der PPA. Das Erkrankungsalter von FTD-Patienten liegt meist zwischen dem 45. und dem 65. Lebensjahr [2].

Die behaviorale Variante der FTD (bvFTD) stellt die häufigste klinische Präsentation einer FTLD dar. Die Erstsymptome sind häufig subtil, die Patienten zeigen fast immer eine Anosognosie für die leitsymptomatischen Verhaltensänderungen, welche von Angehörigen oftmals zunächst als „midlife crisis“ fehlinterpretiert werden. Diese Präsentation resultiert aus den zentralen bvFTD-Symptomen, die auch die Grundlage der Diagnosekriterien nach Rascovsky bilden: sog. „Plussymptome“ wie soziale Disinhibition, perseverierende, stereotype oder zwanghaft, ritualisierte Verhaltensmuster oder Veränderungen im Essverhalten (insbesondere ein gesteigerter Konsum von Süßigkeiten). Andererseits kommt es häufig bei denselben Patienten auch zu Minussymptomen wie Apathie, Antriebsarmut oder Verlust von Empathie [22]. Neben den Verhaltenssymptomen finden sich markante Störungen exekutiver Funktionen trotz relativ intakter Gedächtnisleistungen und visuell-räumlicher kognitiver Funktionen [28]. Die Diagnosekriterien einer „wahrscheinlichen bvFTD“ fordern zudem das Vorhandensein einer Alltagsrelevanz der genannten Symptome, eine Verschlechterung in Bezug auf ein prämorbides Funktionsniveau sowie den Nachweis einer frontotemporalen Atrophie bzw. eines entsprechenden Hypometabolismus in der funktionellen zerebralen Bildgebung (FDG-PET; [28]).

**Figure 3:**
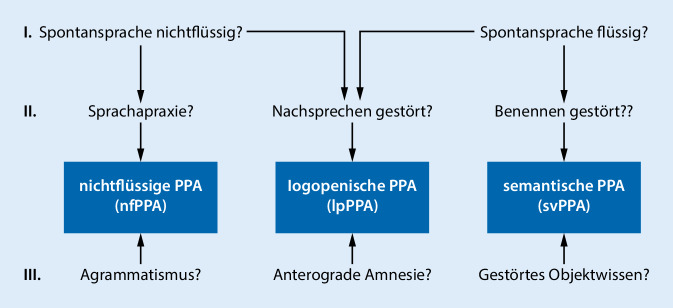

Das zweithäufigste klinische Korrelat der FTD stellen die Sprachvarianten dar, die sog. primär progressiven Aphasien (PPAs; [13]). Das klinische Bild von Patienten mit einer PPA ist gekennzeichnet durch eine langsam progrediente Sprachstörung als vorherrschendes Symptom, welches hauptverantwortlich für die Einschränkungen der Alltagsfunktionalität ist [13]. Anhand der klinischen Präsentationen der PPA werden drei Subtypen unterschieden (Abb. 3): Bei der nichtflüssigen PPA (nfPPA) finden sich eine angestrengte, nichtflüssige Sprachproduktion, eine Sprechapraxie und grammatikalische Defizite mit phonematischen Paraphasien. Bei der semantischen Variante (svPPA) zeigt sich eine flüssige, jedoch inhaltsleere Sprache mit Störungen der Semantik, also der „Wortbedeutung“. Daraus resultieren Benennstörungen, semantische Paraphasien und umständliche Umschreibungen in der Spontansprache. Der dritte Subtyp, die lpPPA ist charakterisiert durch Wortfindungsstörungen und eine reduzierte Sprachproduktion bei relativ gut erhaltener Phonologie und Syntax [13]. Während die nichtflüssige PPA und die semantische PPA meist durch eine FTLD versursacht sind, handelt es sich bei der lpPPA in den meisten Fällen um eine Variante der Alzheimer-Krankheit (s. oben; [24]). Obwohl Sprachstörungen dominieren, lassen sich die drei PPA-Varianten häufig klinisch markant differenzieren (Abb. 3).

### Vaskuläre Demenz

Die vaskuläre Demenz wird in einigen epidemiologischen Studien als häufige Ursache einer Demenz bei jüngeren Menschen angegeben [14, 16]. In diesen Studien verbirgt sich hinter dem Begriff der vaskulären Demenz jedoch eine sehr heterogene Gruppe ätiologisch unterschiedlicher Erkrankungen. Im engeren Sinn versteht man unter einer vaskulären Demenz ein subkortikales demenzielles Syndrom infolge einer zerebralen Mikroangiopathie [30]. Klinisch imponiert eine psychomotorische Verlangsamung mit einer Störung des Abrufens bereits erlernter Gedächtnisinhalte sowie reduzierter Verarbeitungsgeschwindigkeit und Aufmerksamkeit. Die Patienten wirken im Gegensatz zu Alzheimer-Patienten im klinischen Alltag umständlich, leicht ablenkbar und wenig fokussiert. Die zerebrale Mikroangiopathie ist vor dem 65 Lebensjahr sehr selten und deshalb auch ein seltener Grund für eine früh beginnende Demenz. Im weiteren Sinn werden jedoch auch strategisch lokalisierte embolische Infarkte (z. B. in Thalamus, Fornix oder Hippokampus), die zerebrale Amyloidangiopathie, primäre und sekundäre zerebrale Vaskulitiden sowie genetische Erkrankungen wie die zerebrale autosomal-dominante Arteriopathie mit subkortikalen Infarkten und Leukenzephalopathie (CADASIL) zur vaskulären Demenz gezählt. Gemäß der aktuellen S3-Leitlinie stellt für die Diagnose einer vaskulären Demenz das Vorhandensein vaskulärer zerebraler Läsionen und ein zeitlicher Zusammenhang mit dazu passenden kognitiven Funktionsstörungen das dominante diagnostische Kriterium dar [9]. Die oben genannten, heterogenen Ursachen der vaskulären Demenzen sind für sich allein genommen selten, machen in ihrer Summe mit etwa 18 % jedoch einen relativ hohen Anteil der früh beginnenden Demenzen aus [14, 31].

### Sekundäre Demenzen

Unter dem Oberbegriff „sekundäre Demenzen“ werden alle demenziellen Syndrome zusammengefasst, die nicht Folge einer primär neurodegenerativen Erkrankung sind und nicht zu den vaskulären Demenzen zählen. Die kognitiven Störungen können dabei Folge einer sekundären zerebralen Funktionsstörung oder einer sekundären Neurodegeneration sein. Sekundäre Demenzen sind bei jungen Menschen prozentual deutlich häufiger als bei älteren Menschen. Sie machen in ihrer Gesamtheit bei Patienten <65 Jahre knapp ein Drittel aller Fälle aus, bei Patienten <35 Jahre stellen sie sogar die häufigste Ursache einer Demenz dar [14, 18, 31]. Das Feld der Differenzialdiagnosen ist sehr breit: Erkrankungen, die sich mit einem sekundären demenziellen Syndrom manifestieren, umfassen verschiedene Infektionskrankheiten, autoimmunvermittelte Erkrankungen, metabolische und hereditäre Erkrankungen, ethyltoxische oder traumatische Hirnschäden. Einige der sekundären Demenzen sind sehr gut behandelbar und sollten daher nicht übersehen werden. Aus diagnostischer Sicht ist in diesem Zusammenhang das Konzept der Demenz-Plussyndrome von praktischer Bedeutung. Bei vielen der genannten Erkrankungen handelt es sich um Systemerkrankungen, bei denen das demenzielle Syndrom nicht die einzige Krankheitsmanifestation darstellt, sondern lediglich eines von mehreren Symptomen. Bei den Plussymptomen kann es sich sowohl um andere neurologische (z. B. motorische) Symptome oder um Symptome infolge einer Beteiligung anderer Organsysteme handeln [31].

## Diagnostisches Vorgehen

**Figure 4:**
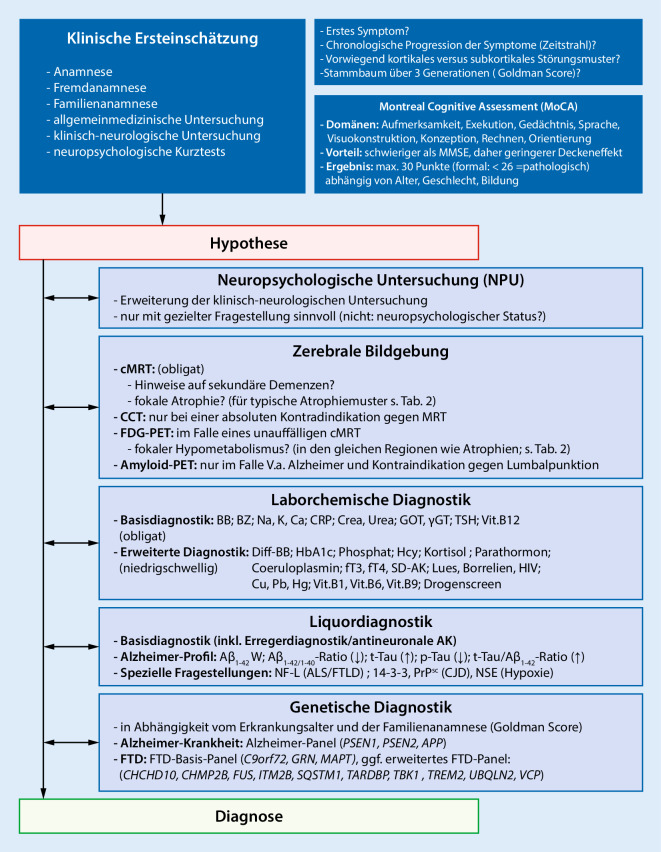

Die korrekte Diagnose bei jüngeren Patienten mit einer Demenz erfordert ein strukturiertes diagnostisches Vorgehen. In Abwesenheit spezifischer Biomarker für den Großteil der möglichen zugrunde liegenden Erkrankungen basiert die Diagnose primär auf den klinischen Symptomen. Die Diagnostik bei jungen Patienten mit Demenz sollte daher hypothesengeleitet erfolgen, d. h. nach einer präzisen klinisch-syndromalen Zuordnung der Symptome (Abb. 4).

### Klinischer Eindruck

Die Erstvorstellung erfolgt in den meisten Fällen zur Abklärung subjektiv empfundener kognitiver Funktionsstörungen, die entweder der betroffenen Person selbst oder Angehörigen aufgefallen sind. Von größter differenzialdiagnostischer Bedeutung ist die Identifizierung des zuerst aufgetretenen Krankheitssymptoms sowie dessen zeitlicher Verlauf. Aufgrund der Art der vorhandenen kognitiven Störungen oder dem Vorliegen einer Anosognosie ist die Anamnese der betroffenen Person in vielen Fällen nicht ausreichend und eine ergänzende strukturierte Fremdanamnese unabdingbar; gleiches gilt für die Familienanamnese.

Auch wenn die Einteilung aus neuropathologischer und neuropsychologischer Sicht stark vereinfacht erscheint, ist eine initiale Einteilung der kognitiven Funktionsstörung in *kortikale *„Werkzeugstörungen“ oder *subkortikale* kognitive Störungen zur Planung des diagnostischen Vorgehens empfehlenswert. Zu den kortikalen neurokognitiven Funktionen zählen Gedächtnis, Sprache, Praxie und Visuokonstruktion. Störungen in diesen Domänen sind typische Symptome der Alzheimer-Krankheit oder der FTLD. Den kortikalen Symptomen gegenüberzustellen sind subkortikale Störungen [15]. Hierzu zählt in erster Linie die psychomotorische Verlangsamung. Das Abrufen bereits erlernter Gedächtnisinhalte ist erschwert, Verarbeitungsgeschwindigkeit und Aufmerksamkeitsleistungen sind reduziert. Patienten mit subkortikalen Störungen wirken im Kontakt unkonzentriert, umständlich, ohne Fokus auf das Wesentliche, fahrig und leicht ablenkbar. Typisches Beispiel für eine subkortikale Demenz ist die zerebrale Mikroangiopathie, aber auch viele sekundäre Demenzen zeigen ein subkortikales Minderleistungsprofil.

Bei jedem Patienten mit Demenzverdacht sollte bereits bei der Erstdiagnose eine standardisierte Quantifizierung der kognitiven Leistungseinbußen erfolgen. Es stehen verschiedene zeitökonomische Tests zur Verfügung, die jeweils mit Vor- und Nachteilen behaftet sind. In der Ersteinschätzung ist ein Multidomänen-Screeningtest sinnvoll [5], z. B. der Mini-Mental State Test (MMST; [11]) oder das Montreal Cognitive Assessment (MoCA; [25]). Der MMST ist weltweit am meisten verbreitet [11]. Für die Frühdiagnose besteht aber ein erheblicher Deckeneffekt (zu einfache Items). Zudem werden v. a. subkortikale Domänen oder Exekutivfunktionen nicht ausreichend erfasst [5]. Das MoCA hat ein ähnliches Design und die gleiche maximal erreichbare Gesamtpunktzahl (30 Punkte) wie der MMST. Es wurde jedoch primär als spezifisches Screeninginstrument zur Detektion leichtgradiger kognitiver Störungen entwickelt, die einzelnen Aufgaben sind schwieriger und die Auswahl kognitiver Domänen breiter gewählt. Dadurch fällt der Deckeneffekt des Testes deutlich geringer aus und das Spektrum der detektierbaren Differenzialdiagnosen ist insbesondere bei jüngeren Patienten breiter [5]. Ein Ergebnis von 26 oder mehr Punkten gilt als normal [25]. Zahlreiche Validierungsstudien zeigen jedoch, dass dieser Cut-off-Wert in vielen Fällen zu streng gewählt wurde. Eine rezente Validierungsstudie der offiziellen deutschen Übersetzung des MoCA demonstrierte eine Spannweite des Cut-off-Wertes von 17 bis 26 Punkten in Abhängigkeit von Geschlecht, Alter und Bildung, die anstelle des starren 26-Punkte-Wertes genutzt werden sollte [33].

Neben der Anamnese ist eine ausführliche medizinische und klinisch-neurologische Untersuchung erforderlich, um systemische oder neurologische Demenz-Plussymptome als Hinweise auf bestimmte Ursachen einer sekundären Demenz nicht zu übersehen. Tab. 1 zeigt eine Übersicht der wichtigsten Differenzialdiagnosen früh beginnender Demenzen mit v. a. internistischen oder klinisch-neurologischen „red flags“ bzw. typischen Ergebnissen der laborchemischen oder bildgebenden Diagnostik. Es ist von entscheidender Bedeutung, potenziell reversible und behandelbare Ursachen nicht zu übersehen. Diese reichen vom Normaldruckhydrozephalus, welcher bereits ab dem 40. Lebensjahr auftreten kann, über die Alkoholkrankheit und Hypovitaminosen bis hin zu den autoimmunvermittelten oder infektiösbedingten Enzephalitiden sowie den hereditären Stoffwechselstörungen. Gerade im vergangenen Jahrzehnt haben die Autoimmunenzephalitiden zunehmend an Bedeutung gewonnen. Durch die Entdeckung spezifischer Autoantikörper gegen neuronale Antigene im ZNS gelang die Klassifizierung spezifischer Krankheitsentitäten. Im Erwachsenenalter neu auftretende kognitive Defizite sind eine typische Präsentation der limbischen Enzephalitiden, meist charakterisiert durch episodische Gedächtnisstörungen, epileptische Anfälle, bilaterale T2/FLAIR-Hyperintensitäten temporomesialer Strukturen im MRT, bitemporale epilepsietypische Potenziale im EEG und den laborchemischen Nachweis spezifischer Autoantikörper im Liquor oder v. a. im Serum (Tab. 1).

**Table 1:** 

Gruppe	Diagnose	„Red flags“ (Symptome/Tests)	
Primär neurodegenerative Erkrankungen	Alzheimer-Krankheit	Anterograde mnestische Defizite	
	FTLD (inkl. PSP, CBD), ALS	Paresen, Atrophien, PBZ	
	PDD, LBD, MSA	Parkinson-Symptome, zerebelläre Symptome, subkortikales Störungsmuster	
	Creutzfeld-Jakob-Krankheit (sCJD)	Myoklonus, Ataxie, PBZ, Mutismus	
Vaskuläre Demenz	Zerebrale Mikroangiopathie	Subkortikales Störungsmuster	
	Multiinfarktsyndrom	MRT: strategische Infarkte	
	Zerebrale Vaskulitis	Hirninfarkte; Labor: entz. Liquor MRT: Gefäßkaliberirregularitäten	
Hydrozephalus	Normaldruckhydrozephalus (NPH)	Inkontinenz, Gangstörung, subkortikales Störungsmuster; MRT: Evans-Index >0,3	
Trauma	Chronisch-traumatische Enzephalopathie	Positive Trauma‑/Sportanamnese; MRT: zerebrale Mikroscherverletzungen	
Ernährung/Toxische Substanzen	Alkoholkrankheit	Desorientiertheit, Augenmuskelparesen, Ataxie	
	Vitaminmangel	Vitamin B1, B3, B6, B9, B12, D	
	Dauerhafte Pharmakotherapie	Trizyklika, Lithium, Valproat, Antipsychotika	
Metabolisch/Endokrinologisch	Hepatopathie	Flapping-Tremor, Labor: Leberwerte (v. a. NH_3_)	
	Nephropathie	Labor: Nierenretentionsparameter (v. a. Urea)	
	Schilddrüsenfunktionsstörung	Hypothyreose, seltener Hyperthyreose	
Autoimmunvermittelte bzw. chronisch-entzündliche ZNS-Erkrankungen	Multiple Sklerose (MS)	Fokal-neurologische Defizite Labor: entz. Liquor; MRT: MS-Läsionen	
	Neuromyelitis optica (NMOSD)	Optikusneuritis, transverse Myelitis Labor: entz. Liquor; Anti-AQP4, MOG-AK	
	Limbische Enzephalitis – Autoimmunenzephalitis – Paraneoplastische Enzephalitis	Epileptische Anfälle, psychotische Symptome Labor: entz. Liquor; Anti-LGI1, CASPR2, GAD, AMPAR, GABA_B_R, Hu, Ma2-AK i.S./L. MRT: temporomesiale T2-Hyperintensität EEG: bitemporale epilepsietypische Potenziale	
	SREAT (Hashimoto-Enzephalitis)	Epileptische Anfälle, Myoklonus, psychotische Symptome; Labor: Anti-TPO, TG-AK	
	Sarkoidose	Mediastinale Lymphadenopathie, Arthralgie, Erythema nodosum; Liquor: Schrankenstörung	
Infektionskrankheiten	HIV	Opportunistische Infektionen, PNP	
	Neurolues	Tabes dorsalis, oft Koinfektion mit HIV	
	Neuroborreliose	Erythema migrans, Bannwarth-Syndrom	
	Whipple-Krankheit	Diarrhö, Malabsorption, Parkinson-Symptome	
	Herpesenzephalitis	Epileptische Anfälle, Aphasie, Vigilanzminderung	
Leukodystrophien	Metachromatische Leukodystrophie	*ARSA*; AR	Augen‑, Haut-Symptome, epileptische Anfälle, Paraspastik, PBZ, extrapyramidalmotorische Störungen, Kleinhirnsymptome, Bronze-Diabetes MRT: bilateral symmetrisch, konfluierende Leukenzephalopathie
	Adrenoleukodystrophie	*ABCD1*; XR	
	Krabbe-Krankheit	*GALC*; XR	
	Alexander-Krankheit	*GFAP*; AD	
	CADASIL	*NOTCH3*; AD	
	Zerebrotendinöse Xanthomatose	*CYP27A1*; AR	
	Adulte auto.-dom. Leukodystrophie	*LMNB1*; AD	
	Adulte Polyglucosankörpererkrankung	*GBE1*; AR	
	Adulte Leukoenzephalopathie mit axonalen Sphäroiden	*CSF1R*; AD	
	Fragile-X-Tremor-Ataxie-Syndrom	*FMR1*; XD	
Andere hereditäre Erkrankungen	Huntington-Krankheit	*HTT*; AD	Choreatiforme Störung
	Niemann-Pick-Krankheit Typ C	*NPC1*; AR	Ataxie, Slow-Blicksakkaden, Kataplexie, Splenomegalie
	Fabry-Krankheit	*GLA*; XR	Parästhesien, Angiokeratome, Hirninfarkte
	Wilson-Krankheit	*ATP7B*; XR	Extrapyramidalmotorische Störungen, Hepatopathie
	Mitochondriopathien	Myopathie, epileptische Anfälle, PNP	
	Spinozerebelläre Ataxie	Zerebelläre Symptome	
	Hereditäre spastische Paralyse	Paraparese, PBZ, Inkontinenz	
	Neurodegeneration mit Eisenspeicherung	Extrapyramidalmotorische Störungen MRT: erhöhte Eisenspiegel in Basalganglien	
	Neuroakanthozytose	Dyskinesien; Labor: Akanthozyten	

*FTLD* frontotemporale Demenz, *PSP* progressive supranukläre Blickparese, *CBD* kortikobasale Degeneration, *ALS* amyotrophe Lateralsklerose, *PDD* Parkinson-Demenz, *LBD* Lewy-Lörperchen-Demenz, *MSA* Multisystematrophie, *sCJD* sporadische Creutzfeld-Jakob-Krankheit, *PBZ* Pyramidenbahnzeichen, *MRT* Magnetresonanztomographie, *NH*_*3*_ Ammoniak, *Urea* Harnstoff, *AQP4* Aquaporin 4, *MOG* Myelin-Oligodendrozyten-Glykoprotein, *AK* Antikörper, *LGI1* „leucine-rich glioma-inactivated protein 1“, *CASPR2* „contactin-associated protein-like 2“, *GAD* „glutamate decarboxylase“, *AMPAR* ein Glutamatrezeptor, *GABA*_*B*_*R* γ-Aminobuttersäure-Rezeptor B, *i.S./L.* im Serum und/oder Liquor, *EEG* Elektroenzephalographie, *SREAT* steroidresponsive Enzephalopathie mit assoziierter Autoimmunthyroiditis, *TPO* Thyreoperoxidase, *TG* Thyreoglobulin, *HIV* „human immunodeficiency virus“, *PNP* Polyneuropathie, *AD* autosomal-dominant, *AR* autosomal-rezessiv, *XR* X-chromosomal-rezessiv, *XD* X-chromosomal-dominant

### Ausführliche neuropsychologische Testung

Eine ausführliche neuropsychologische Untersuchung (NPU) ist ein integraler Bestandteil des diagnostischen Assessments und sollte als Erweiterung der klinischen Untersuchung zur Einschätzung höherer kognitiver Funktionen eingesetzt werden [36]. Im Gegensatz zu neuropsychologischen Kurztests liefert eine NPU qualitative und quantitative Information über die Funktion der wesentlichen kognitiven Domänen (komplexe Aufmerksamkeit, exekutive Funktionen, Lernen und Gedächtnis, Sprache, perzeptuell-motorische Fähigkeiten und soziale Kognition) sowie deren Einfluss auf mögliche individuelle Einschränkungen im Alltag der Patienten [36]. Den verschiedenen kognitiven Funktionen liegt die Integrität ausgedehnter, neuronaler Netzwerke zugrunde, weshalb bestimmte Konstellationen kognitiver Störungen auf eine Pathologie in spezifischen Hirnregionen hinweisen können. Es sollte jedoch nicht bei allen Patienten niedrigschwellig eine wenig fokussierte neuropsychologische Testung erfolgen. Stattdessen ist eine spezifische, hypothesengeleitete Fragestellung, basierend auf dem klinischen Eindruck unabdingbar, um die Effektivität der neuropsychologischen Untersuchung zu erhöhen [17].

### Zerebrale Bildgebung

Jeder junge Patient mit neu aufgetretenen kognitiven Störungen muss eine zerebrale Bildgebung erhalten. Die Magnetresonanztomographie (MRT) ist der Computertomographie (CT) aufgrund der größeren Genauigkeit bei jung Erkrankten vorzuziehen. Primär geht es dabei um den Ausschluss symptomatischer Ursachen der Demenz (z. B. Tumoren, entzündliche oder vaskuläre Läsionen, siehe Tab. 1). Über die Hälfte der sekundären oder reversiblen Demenzen mit frühem Manifestationsalter können mithilfe der strukturellen MRT diagnostiziert werden [6]. Bei Patienten mit vorwiegend kortikalen Symptomen stellt sich zudem die Frage nach spezifischen Atrophiemustern (Tab. 2). Eine Indikation für eine MRT zur routinemäßigen Verlaufskontrolle besteht im Regelfall nicht, kann bei atypischen Verläufen oder diagnostischer Unsicherheit aber sinnvoll sein.

**Table 2:** 

Klinisches Syndrom	Pathologie	MRT-Atrophie
Amnestisches Syndrom	AD >> FTLD	Medial temporal
Posteriore kortikale Atrophie	AD >> FTLD	Okzipital/parietal/temporal
Verhaltensauffälligkeiten	FTLD >> AD	Frontal/anterior temporal
Nichtflüssige PPA (nfPPA)	FTLD >> AD	Posterior frontoinsular (links)
Semantische PPA (svPPA)	FTLD	Anterior temporal (links > rechts)
Logopenische PPA (lpPPA)	AD > FTLD	Temporal/parietal (links)

Die strukturelle MRT ist in frühen Stadien primärer Demenzen oft wenig wegweisend. In Einzelfällen ist es deshalb sinnvoll, die strukturelle MRT um eine funktionelle Bildgebung zu ergänzen (z. B. Fluordesoxyglucose(FDG)-Positronenemissionstomographie [PET] zum Nachweis eines regionalen Hypometabolismus, Dopamintransporter(DAT)-Scan zum Nachweis eines striatalen Dopamindefizits [bei unklaren motorischen Symptomen] oder Amyloid-PET zum Nachweis regionaler Aβ-Ablagerungen). Eine funktionelle Bildgebung ist nicht indiziert, wenn sich im konventionellen MRT bereits ein klassisches Atrophiemuster zeigt.

### Laborchemische Untersuchungen und Liquordiagnostik

Durch laborchemische Untersuchungen können potenziell reversible Ursachen einer Demenz diagnostiziert werden. Dabei sollte die Demenzbasisdiagnostik, welche für alle Patienten mit einer Demenz unabhängig vom Erkrankungsalter gilt, bei jungen Menschen niederschwellig um weiterführende Serum- bzw. Plasmauntersuchungen ergänzt werden (Abb. 4; [9]).

Eine Liquordiagnostik wird bei jungen Menschen mit einer Demenz empfohlen [9]. Sie ist wichtig für die Identifizierung potenziell behandelbarer Infektionen oder autoimmunvermittelter Ursachen. Zudem erfolgt die Bestimmung der Konzentration spezifischer Proteine, die wichtige Hinweise auf das Vorhandensein bestimmter neurodegenerativer Erkrankungen liefern kann. Etablierte Biomarker der Alzheimer-Krankheit sind die verschiedenen Formen der Proteine Amyloid-β(Aβ) und Tau. Von ersterem sollten die Aβ_1–42_-Isoform sowie die Aβ_1–42_/Aβ_1–40_-Ratio bestimmt werden, von letzterem sowohl das Gesamtprotein (t-Tau) als auch die Threonin(T)-181- oder T‑231-phosphorylierte Form (p-Tau; [27]). Das typische Alzheimer-Liquorbiomarkerprofil, welches Bestandteil der Diagnosekriterien der Alzheimer-Krankheit ist, umfasst eine Reduktion der Konzentration von Aβ_1–42_, eine Reduktion der Aβ_1–42_/Aβ_1–40_-Ratio sowie eine Erhöhung von t‑Tau und p‑Tau. Die Erhöhung von t‑Tau für sich allein genommen ist ein molekularer Biomarker für den Untergang von Nervenzellen, ohne Spezifität für die zugrunde liegende Ursache. Ähnliches gilt für die Proteine 14-3‑3, neuronenspezifische Enolase (NSE) und Neurofilament-Leichtketten (NF-L), die insbesondere bei Patienten mit Verdacht auf Creutzfeld-Jakob-Krankheit (14-3-3), posthypoxischen Hirnschäden (NSE) oder der amyotrophen Lateralsklerose (ALS) und der FTLD (NF-L) bestimmt werden und in Einzelfällen einen diagnostischen Zusatznutzen bieten können. Auch der Nachweis fehlgefalteten Prionproteins (PrP^Sc^) im Liquor bei Patienten mit sporadischer Creutzfeld-Jakob-Krankheit ist möglich [32].

### Molekulargenetik

In begründeten Verdachtsfällen, insbesondere bei jungen Menschen mit positiver Familienanamnese oder typischen Symptomkonstellationen, kann eine molekulargenetische Diagnostik zum Nachweis krankheitsverursachender Mutationen erfolgen. Monogenetisch verursachte Formen der Alzheimer-Krankheit sind jedoch selten: Weniger als 1 % aller Patienten mit einer Alzheimer-Krankheit weisen eine autosomal-dominant vererbte Mutation in einem der drei häufigsten Gene *PSEN1, PSEN2* und *APP* auf, die Penetranz bei vorhandener Mutation beträgt jedoch nahezu 100 %. Monogenetische autosomal-dominante Mutationen sind wahrscheinlich ursächlich in ca. 10–30 % aller FTD-Fälle. Monogenetische Mutationen sind in 90 % der Fälle in einem der drei Gene *MAPT, GRN* und *C9orf72* lokalisiert, daneben existiert noch eine Vielzahl weiterer Gene, die zusammen jedoch nur knapp 5 % der monogenetisch determinierten FTLD ausmachen (Abb. 4). Die Penetranz der FTLD-verursachenden Genmutationen ist variabler.

Allgemein akzeptierte Algorithmen zur genetischen Testung bei Patienten mit einer primär neurodegenerativen Demenz liegen derzeit nicht vor [12]. Eine Testung kommt bei jungem Erkrankungsalter und mehreren betroffenen Familienangehörigen in Betracht. Bei der Erhebung der Familienanamnese sollte ein Stammbaum über drei Generationen erhoben werden. Mithilfe des modifizierten Goldman-Scores lässt sich das Ergebnis der Familienanamnese bzw. Stammbaumanalyse klassifizieren [3] und anhand dessen die Wahrscheinlichkeit für das Vorliegen einer krankheitsverursachenden Mutation besser abschätzen (Tab. 3).

**Table 3:** 

Score	Beschreibung
*Score 1* Autosomal-dominante Vererbung	≥3 betroffene Verwandte in 2 Generationen, wobei eine der 3 betroffenen Personen Verwandter 1. Grades beider anderen ist
*Score 2* Familiäre Häufung	≥3 betroffene Verwandte, ohne dass die Kriterien für Score 1 erfüllt sind
*Score 3 (a/b)* Ein betroffener Verwandter 1. Grades	Manifestationsalter des betroffenen Verwandten: a) <65 Jahre b) ≥65 Jahre
*Score 4* Keine relevante Familienanamnese	Die Familienanamnese erfüllt nicht die Kriterien für Score 1 bis 3 oder die Familienanamnese ist unbekannt

## Fazit für die Praxis

Früh beginnende Demenzen umfassen alltagsrelevante kognitive Störungen mit Beginn zwischen dem 18. und 65. Lebensjahr. Obwohl Demenzen bei älteren Menschen deutlich häufiger sind, macht der Anteil jüngerer Patienten in spezialisierten Gedächtnisambulanzen bis zu einem Drittel der Fälle aus. Das differenzialdiagnostische Krankheitsspektrum ist deutlich breiter. Die häufigste Ursache einer früh beginnenden Demenz ist die Alzheimer-Krankheit, die sich im Gegensatz zu älteren Patienten jedoch in bis zu zwei Drittel der Fälle als „atypische“ Varianten (PCA oder lvPPA) präsentiert. Die zweithäufigste Ursache ist die FTLD (als Verhaltensvariante oder PPA). Je jünger das Erkrankungsalter, desto höher die Wahrscheinlichkeit, dass es sich um eine sekundäre Demenz handelt. Um potenziell reversible Ursachen nicht zu übersehen und die Vielzahl klinischer Syndrome der primär neurodegenerativen Erkrankungen zu differenzieren, ist ein systematisches diagnostisches Vorgehen unabdingbar. Die Diagnostik sollte jedoch hypothesengeleitet sein und auf klinisch-syndromatologischen Einschätzungen beruhen, um unnötige und nicht indizierte Untersuchungen zu vermeiden.
